# A Novel Large Animal Model of Thrombogenic Coronary Microembolization

**DOI:** 10.3389/fcvm.2019.00157

**Published:** 2019-11-05

**Authors:** Olympia Bikou, Serena Tharakan, Kelly P. Yamada, Taro Kariya, Alexandra Gordon, Satoshi Miyashita, Shin Watanabe, Yassine Sassi, Kenneth Fish, Kiyotake Ishikawa

**Affiliations:** Cardiovascular Research Center, Icahn School of Medicine at Mount Sinai, New York, NY, United States

**Keywords:** animal model, coronary microembolization, no-reflow, thrombus injection, large animal, ischemia reperfusion, coronary thromboembolism, myocardial infarction

## Abstract

Coronary microembolization is one of the main causes of the “no-reflow” phenomenon, which commonly occurs after reperfusion of an occluded coronary artery. Given its high incidence and the fact that it has been proven to be an independent predictor of cardiac morbidity and mortality, there is an imperative need to study its underlying mechanisms and pathophysiology. Large animal models are essential to perform translational studies. Currently there is no animal model that recapitulates a clinical scenario of thrombogenic microembolism with preceding myocardial ischemia. Therefore, the goal of this study was to develop and characterize a novel pig model of coronary microembolization using autologous thrombus injection (CMET). Twenty-three pigs underwent myocardial infarction through percutaneous balloon occlusion of the left anterior descending artery (LAD). Each animal was enrolled in one of two groups: (1) the CMET group, in which the LAD occlusion was followed by delivery of autologous clotted blood in the LAD (distal to the balloon occlusion) and reperfusion; (2) the ischemic reperfusion (I/R) group, in which the LAD ischemia was followed by reperfusion. Surviving animals underwent functional and morphological characterization at 1-week post-procedure. Three sham operated animals were used as a control. CMET resulted in impaired left ventricular function compared to I/R pigs at 1 week. Three-dimensional echocardiography demonstrated reduced ejection fraction in the CMET group (CMET vs. I/R: 35.6 ± 4.2% vs. 47.6 ± 2.4%, *p* = 0.028). Invasive hemodynamic measurements by Swan-Ganz and left ventricular pressure-volume catheters revealed that CMET impaired left ventricular contractility and diastolic function. This was confirmed by both load-dependent indices including cardiac output (CMET vs. I/R: 2.7 ± 0.2 l/min, vs. 4.0 ± 0.1 l/min, *p* = 0.002) and load independent indices including preload-recruitable stroke work (CMET vs. I/R: 25.8 ± 4.0 vs. 47.5 ± 6.5 mmHg, *p* = 0.05) and end-diastolic pressure-volume relationship (slope, 0.68 ± 0.07 vs. 0.40 ± 0.11 mmHg/ml, *p* = 0.01). Our unique closed-chest model of coronary microembolization using autologous thrombus injection resembles the clinical condition of thrombogenic coronary microembolization in I/R injury. This model offers opportunities to conduct translational studies for understanding and treating coronary microembolization in myocardial infarction.

## Introduction

Myocardial ischemia as the result of myocardial infarction (MI) is still a leading cause of death (1). Despite significant progress in the treatment of MI, in-hospital mortality and post-MI morbidity remain very high (2). This is partly attributed to the impact of ischemia/reperfusion (I/R) injury on the myocardium and the coronary microcirculation (3, 4). The coronary “no-reflow” phenomenon is a common manifestation of severe I/R injury. “No-reflow” was first described by Krug et al. (5) and Kloner et al. (6). It is defined as the inability to reperfuse part of the myocardium despite restoration of epicardial coronary flow following MI (7). One of the main contributors to the “no-reflow” phenomenon is coronary microvascular obstruction induced by coronary microembolization (CME) of atherosclerotic plaque debris and thrombotic materials.

The reported incidence of CME during percutaneous coronary intervention varies between 0 and 70%, depending on the method of assessment (8). Numerous studies established evidence that “no-reflow” and CME in humans are associated with worse outcomes and poor prognosis (7, 9–11). These facts prompted the development of animal models to study the pathophysiology of CME. CME has been induced in various small and large animal models, mainly through the injection of microspheres in the coronary circulation. However, microspheres are chemically inert and do not adequately recreate the clinical situation in MI patients. The actual microemboli consist of clotted blood and atherosclerotic plaque debris. In addition to the physical obstruction, the biological contents of the emboli also contribute to “no-reflow” by initiating and/or enhancing thrombogenic, inflammatory and vasoconstriction reactions (8).

The current guidelines for experimental models of myocardial ischemia and infarction recommend large animals for studying periprocedural infarction (12). Nevertheless, to our knowledge, a large animal model that resembles thrombogenic CME after reperfusion does not exist.

Therefore, the aim of this study was to develop a new large animal model of thrombogenic CME using autologous thrombus injection (CMET). We used a percutaneous approach to induce acute anterior MI in pigs. Thrombogenic CME was modeled by injecting autologous thrombus, consisting of clotted blood, into the occluded coronary artery (left anterior descending, LAD). After an observation period of 1 week, we characterized this model functionally and morphologically.

## Materials and Methods

### Animal Care

The experimental protocols were approved by the Institutional Animal and Use Committee at the Icahn School of Medicine at Mount Sinai. The study was performed in compliance with the *Guide for the Care and Use of Laboratory Animals*. The animals were acclimatized at the Center for Comparative Medicine and Surgery at Mount Sinai before being enrolled in experiments.

### Experimental Preparation

Twenty-six female Yorkshire pigs, with body weights of 38–43 kg were used in this study. The pigs were fasted overnight before the procedure but had free access to drinking water. The animals were sedated by intramuscular injection of Telazol (tiletamine/zolazepam, 8 mg/kg). After intubation and placement of an intravenous catheter, the pigs were ventilated with 100% oxygen. Propofol at a dose of 8–10 mg/kg/h was used in all procedures and intravenous normal saline infusion (0.9% NaCl) was maintained throughout the procedure. The animals were continuously monitored with an electrocardiogram, pulse oximeter, and rectal temperature probe.

### Study Design

**Figure 1:**
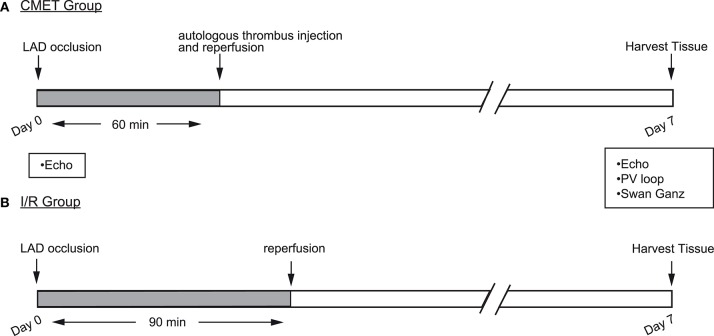
Study design. Myocardial infarction (MI) was induced by occlusion of the mid LAD using an over-the-wire coronary balloon. **(A)** Coronary microembolization using autologous thrombus injection group (CMET): 60 min after LAD occlusion and with the balloon still inflated, autologous thrombus was injected through the balloon wire lumen. After the injection, the balloon was deflated to allow tissue reperfusion. **(B)** Ischemia reperfusion (I/R) group: the coronary balloon was deflated after 90 min to allow tissue reperfusion. In both groups, cardiac functional measurements were performed 1 week after the MI. LAD, left anterior descending artery; Echo, echocardiography; PV, pressure volume.

The aim of this study was to establish and characterize a large animal model of CMET. Each animal was enrolled in one of the three groups: (1) the CMET group, in which the LAD was occluded, followed by delivery of autologous clotted blood distal to the balloon occlusion and reperfusion thereafter (*n* = 14); (2) the I/R group, in which the LAD ischemia was followed by reperfusion (*n* = 9), and (3) the sham group, in which the animals underwent anesthesia without induction of coronary ischemia (*n* = 3). The CMET and I/R animals were clinically observed for 1 week. Echocardiographic assessment was performed before and at 1 week after MI. Functional hemodynamic assessments were made at the 1-week time point. These included: Swan-Ganz measurements and left ventricular pressure-volume measurements. A subset of animals was euthanized immediately after the measurements and the heart was harvested for infarct size assessment. The sham animals underwent echocardiography, hemodynamic measurements and euthanasia on the same day. A summary of the experimental design is shown in the Figure 1.

### MI Creation

After the baseline evaluation of cardiac function by echocardiography, boluses of atropine (0.05 mg/kg) and amiodarone (2 mg/kg) were given intravenously and intramuscularly, respectively. Throughout the procedure a normal saline infusion supplemented with atropine (0.1 mg/kg), amiodarone (2 mg/kg), and potassium acetate (20 mEq) in 1 l bag was continuously infused at a rate of 300 ml/h. Intravascular access was established by percutaneous puncture of the femoral artery under ultrasound guidance. A 7-Fr hockey stick catheter (Cordis) was then advanced to the left coronary artery. After acquisition of the coronary angiogram, a 0.014-inch coronary wire was advanced in the LAD. Over-the-wire coronary balloon (4.0 × 8 mm NC TREK, Abbott) was then advanced to the mid LAD, distal to the first major diagonal branch. The balloon was inflated to 3–4 atm. LAD was occluded for 90 min in the I/R group to induce near transmural infarction. The CMET group received an autologous thrombus injection at 60 min after balloon occlusion, which is the critical period for frequent ventricular arrhythmias in pigs during ischemia. Malignant arrhythmias were immediately treated with direct current cardioversion (150 J) accompanied by chest compression. After recovery, the animals were examined daily. In case of unexpected deaths, the animals were examined by the investigators and a veterinary pathologist.

### Coronary Microembolization Using Autologous Thrombus Injection

After establishing the vascular access in the femoral artery, 6 ml of autologous blood was withdrawn and stored in a sterile glass tube to allow thrombus formation. Heparin was thereafter administered to the animal to maintain an activated coagulation time of 250–300 s. Just prior to injection, the clotted blood was prepared as follows: the clotted blood was carefully taken out of the glass tube and placed on a sterile surface. Approximately 1 cm^3^ of thrombus was cut using a scalpel and transferred into a 3 ml syringe. Another syringe was prepared with 2 ml of X-ray contrast. The air was carefully removed from both syringes and they were connected to a 3-way connector. The content of both syringes was thoroughly mixed until no resistance was encountered upon movement of the syringes. The procedure of the thrombus preparation is shown in Supplemental Figure 1 and Supplemental Movie 1.

At the end of the LAD occlusion time (60 min), with the balloon still inflated, ~1 ml of the thrombus/contrast mixture was injected through the balloon wire lumen. The balloon was slowly deflated beginning ~1 min after injection of the thrombus. After balloon removal the LAD flow was assessed in two different projections (RAO 90° and LAO 30°). The Thrombolysis in Myocardial Infarction (TIMI) flow grade scale was used to assess the blood flow. After the confirmation of stable hemodynamics, the animal was allowed to recover and was examined daily.

### Echocardiography

Prior to every procedure, the animals underwent echocardiographic examination using a Philips iE-33 ultrasound system (Philips Medical Systems, Andover, MA). Two-dimensional (2D) and three-dimensional (3D) measurements were acquired using a multi-frequency imaging transducer (S5 probe and X-3 Probe, respectively). Sub-xiphoidal access was used for the four-chamber view and right parasternal access for the short axis view. Analysis of the echocardiographic data has been described in detail elsewhere (13). Left ventricular volumes and ejection fraction were analyzed from the 3-D images. Body surface area (BSA) was calculated as previously described (14). Volume parameters were divided by the BSA in order to calculate the volume indices.

### Hemodynamic Measurements

A Swan-Ganz catheter (Edwards Lifesciences, Irvine, CA) was proceeded to the main pulmonary artery through the femoral venous access and advanced to each anatomic position in order to acquire the following measurements: pulmonary capillary wedge pressure, pulmonary arterial pressure, and right atrial pressure. The cardiac output was measured in triplicate using the thermodilution method. All measurements were performed after confirmation of hemodynamic stability and during breathhold. A Millar pressure-volume catheter (Millar instruments, Auckland, New Zealand) was inserted from percutaneously established right carotid arterial access. Pressure-volume signals were recorded using iox2 software (Emka technologies, Falls Church, VA). For reducing preload, temporary occlusion of the inferior vena cava was performed using a Fogarty balloon catheter.

### Post-mortem Examination

Seven pigs were euthanized immediately after the echocardiographic and hemodynamic assessment at 1 week. The heart was quickly removed and the great vessels were dissected. The atria were carefully separated from the ventricular chambers. The right ventricular free wall was dissected and each part of the myocardial chamber was weighed separately. The left ventricle was immersed in cold crystalloid cardioplegic solution and sectioned into 5–6 slices in parallel to the short axis. Scar size was quantified by digital planimetry.

### Statistical Analysis

Statistical analysis was performed with commercially available software (Graphpad Prism, version 7). Data are shown as mean ± SEM. The distribution of the data was determined by Shapiro Wilk's or D'Agostino-Pearson omnibus normality test. The *t*-test was used to assess differences between two groups. If the normality test indicated a non-Gaussian distribution, the Mann-Whitney test was used. The two-way repeated measures analysis of variance (ANOVA) was used for testing datasets with two groups and two time points. All three study groups were compared at the 1-week time point using the one-way ANOVA (Gaussian distribution) or Kruskal-Wallis test (when the data distribution was not Gaussian). The mortality calculations were performed using Log-rank (Mantel-Cox) test. A *p*-value <0.05 was considered statistically significant.

## Results

### Coronary Microembolization Using Autologous Thrombus Injection Is Associated With Higher Early Mortality

**Figure 2:**
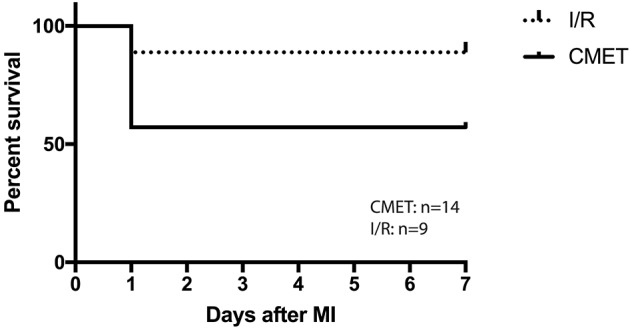
CMET leads to higher mortality. Survival curve of the pigs in I/R and CMET groups. I/R, ischemia/reperfusion; CMET, coronary microembolization using autologous thrombus injection; MI, myocardial infarction.

Twenty-six pigs were initially enrolled in this study. Fourteen pigs underwent the CMET protocol, and nine pigs underwent the ischemia reperfusion (I/R) protocol. Three sham-operated animals were subjected to same functional measurements and euthanized on the same day. Therefore, the sham pigs were not included in the mortality study. One pig in the CMET group and one in the I/R group died during the procedure due to irreversible malignant arrhythmias. Five pigs in the CMET group died within 24 h post-procedure. Overall mortality was 43% in the CMET group and 11% in the I/R group (*p* = 0.11). Mortality after excluding in-procedure deaths was significantly higher in the CMET group compared to the I/R group (*p* = 0.05). Survival curves of the CMET and I/R groups are shown in the Figure 2.

### Coronary Microembolization Leads to Impaired Left Ventricular Systolic and Diastolic Function

**Figure 3:**
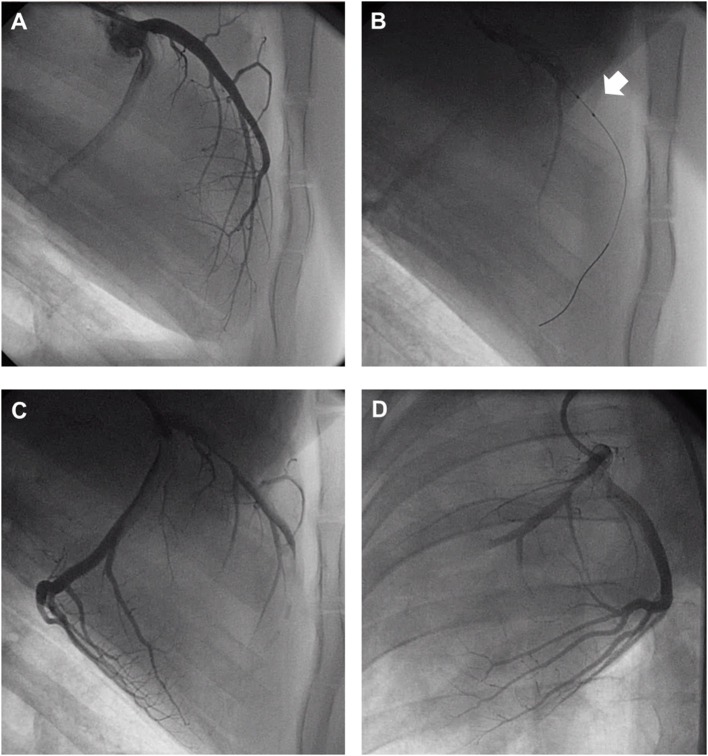
Representative coronary angiogram images of a pig injected with autologous thrombus. **(A)** Left anterior descending artery (LAD) before occlusion. **(B)** LAD during occlusion with an intracoronary balloon (Arrow). **(C)** LAD post-autologous thrombus injection and reperfusion in the RAO 90° and **(D)** LAO 30° projection. LAO, left anterior oblique; RAO, right anterior oblique. Angiogram movies are available in Supplemental Movie 1.

**Figure 4:**
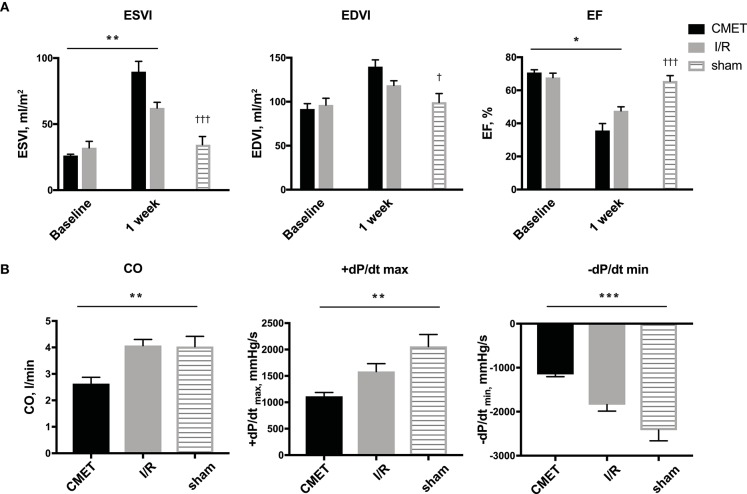
CMET leads to increased left ventricular remodeling and reduced left ventricular function. **(A)** LV volume indices and the EF of the animals before and 1 week after myocardial infarction. CMET leads to significant reduction of LV EF. Measurements were performed using 3D echocardiography, *n* = 8 each for the CMET and I/R group, *n* = 3 for the sham group. Change over time was evaluated by the time × group interaction by repeated measures ANOVA (^*^). Comparison between the study groups (CMET vs. I/R vs. sham) was performed using one-way ANOVA (^†^). **(B)** Load dependent parameters of LV function: cardiac output, maximum and minimum left ventricular dP/dt (dP/dt max and -dP/dt min, respectively), as measured using the pressure volume catheter, *n* = 7 for the CMET, *n* = 8 for the I/R and *n* = 3 for the sham group. *P*-values were determined by the one-way ANOVA. CMET, coronary microembolization using autologous thrombus injection; I/R, ischemia/reperfusion; LV, left ventricular; EDVI, end-diastolic volume index; ESVI, end-systolic volume index; EF, ejection fraction; CO, cardiac output. All data reported as mean ± SEM. ^*^p < 0.05, ^**^p < 0.01, ^***^p < 0.001, ^†^*p* < 0.05, ^†*††*^*p* < 0.001.

Acutely, all the pigs in the CMET group exhibited impaired coronary flow (TIMI grade 0 in 4 pigs, I in 3 pigs, and II in 1 pig). Representative coronary angiogram images of a pig in the CMET Group are shown in the Figure 3 and Supplemental Movie 2. Table 1 summarizes the results of the echocardiographic assessment at baseline and 1 week after MI. Regional wall motion of the anterior wall showed severe hypo- and akinesia in both CMET and I/R Groups. Direct comparison between the groups revealed that CMET aggravates left ventricular remodeling. This was indicated by larger end-diastolic and end-systolic volumes, as assessed by 3D echocardiography (Table 1). The end-systolic volume index increased from 26.1 ± 1.1 to 89.6 ± 7.9 ml/m^2^ (*p* < 0.001) in the CMET group, and from 32.1 ± 4.9 to 62.2 ± 4.3 ml/m^2^ (*p* = 0.002) in the I/R group. The end-diastolic volume index increased from 91.6 ± 6.3 to 139.7 ± 8.0 ml/m^2^ (*p* < 0.001) in the CMET group and from 96.1 ± 7.8 to 118.5 ± 5.3 ml/m^2^ (*p* = 0.06) in the I/R group. Left ventricular ejection fraction was severely impaired in the CMET group compared to the I/R group at the 1-week time point (Figure 4). Heart rate, body weight and body surface area showed no significant differences between the groups (Table 1).

**Table 1:** Variables from non-invasive measurements.

	**Baseline**		**1 week**			
	**CMET (*n* = 8)**	**I/R (*n* = 8)**	**CMET (*n* = 8)**	**I/R (*n* = 8)**	**Sham (*n* = 3)**	***P*-value**
**3D ECHOCARDIOGRAPHY DATA**						
EDV (ml)	75.4 ± 4.5	79.8 ± 6.7	118 ± 7.1	99.4 ± 4.4	83.3 ± 6.6	0.014
ESV (ml)	21.7 ± 1.1	26.6 ± 4.1	75.3 ± 6.1^**^	52.2 ± 3.7	28.7 ± 4.3	<0.001
SV (ml)	53.7 ± 4.2	53.2 ± 3.5	42.7 ± 6.2	47.2 ± 3.0	52.4 ± 5.9	0.549
**OTHER**						
Heart rate (beats/min)	85.0 ± 3.8	80.1 ± 3.6	89.5 ± 2.4	88.8 ± 4.5	99.7 ± 7.9	0.318
Body weight (Kg)	40.4 ± 1.7	40.4 ± 0.9	41.5 ± 1.7	41.1 ± 1.0	41.3 ± 2.0	0.981
BSA (m^2^)	0.83 ± 0.0	0.83 ± 0.0	0.84 ± 0.0	0.84 ± 0.0	0.8 ± 0.0	0.99

*CMET, coronary microembolization using autologous thrombus injection; I/R, ischemia/reperfusion; 3D, 3-dimensional; EDV, end-diastolic volume; ESV, end-systolic volume; SV, stroke volume; BSA, body surface area. Values are means ± SEM. P-value was determined by one-way ANOVA for the 1-week time point*.

** *p < 0.01 for time × group interaction by repeated measures analysis of variance for the CMET vs. I/R group at two time points*.

Impaired cardiac performance of the CMET group was additionally verified by invasive hemodynamic measurements. Cardiac output, as assessed by the thermodilution method, was significantly reduced in the CMET group compared to the I/R group at the 1-week time point. Interestingly, cardiac output of the I/R group was not significantly different from that of the sham group, suggesting compensation of systolic function at 1 week post-MI in I/R group. The maximum left ventricular pressure was significantly lower in the CMET group. Furthermore, maximum left ventricular dP/dt, another parameter of systolic function, was significantly lower in the CMET group compared to the I/R group (Figure 4). Pulmonary capillary wedge pressure was significantly higher in the CMET group (Table 2). Tau and minimum left ventricular dP/dt, as indices of left ventricular relaxation, were also impaired in the CMET group compared to the I/R group without statistical significance in Tau (Table 2 and Figure 4). However, both groups had significantly prolonged Tau compared to the sham animals. For one animal in the CMET group, no invasive measurements were possible due to procedural complications. Therefore, Swan-Ganz and pressure volume measurements of seven CMET animals were included in the analysis.

**Table 2:** Invasive hemodynamic measurements 1 week after myocardial infarction.

	**CMET (*n* = 7)**	**I/R (*n* = 8)**	**Sham (*n* = 3)**	***P*-value**
mAoP (mmHg)	69.1 ± 4.5	92.7 ± 6.7^#^	105.3 ± 9.2	0.007
mPAP (mmHg)	18.4 ± 0.7	16.1 ± 2.0	12.3 ± 2.4	0.157
CO (l/min)	2.7 ± 0.2	4.1 ± 0.2	4.0 ± 0.4	0.002
mean PCWP (mmHg)	10.7 ± 1.0	6.0 ± 1.2	3.0 ± 0.6	0.005
Tau (ms)	61.4 ± 3.2	53.6 ± 2.9	41.3 ± 1.0	0.006
maximum LV pressure (mmHg)	85.0 ± 5.1	109.4 ± 7.9	127.3 ± 12.2	0.007

CMET, coronary microembolization using autologous thrombus injection; I/R, ischemia/reperfusion; mAoP, mean aortic pressure; mPAP, mean pulmonary arterial pressure; CO, cardiac output; PCWP, pulmonary capillary wedge pressure; LV, left ventricular;

# *n = 7. Values are means ± SEM. P-value as determined by 1-way ANOVA or Kruskal-Wallis test*.

### Coronary Microembolization Impairs Left Ventricular Contractility and Stiffness 1 Week After MI

**Figure 5:**
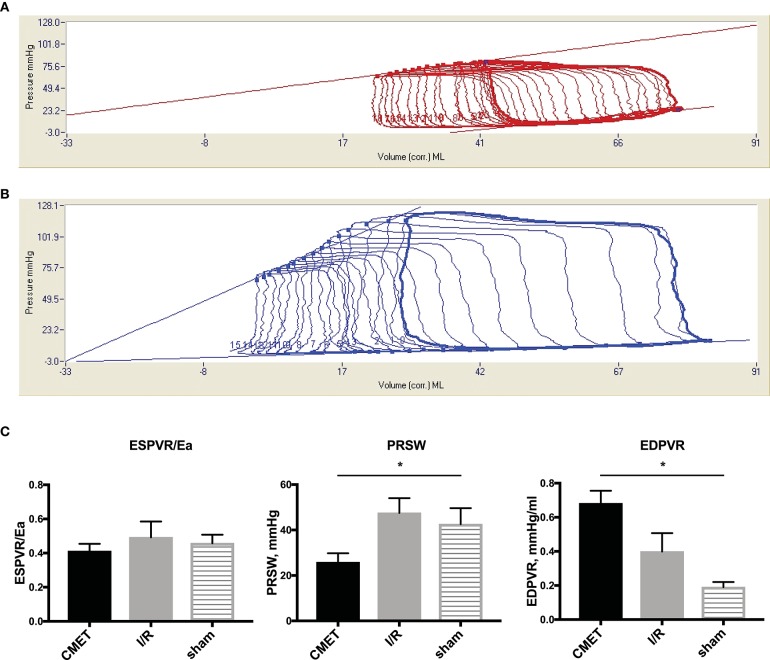
Pressure-volume relationships 1 week after MI. **(A)** Representative LV pressure-volume loops during preload reduction 1 week after CMET and **(B)** after I/R. **(C)** Load independent parameters of LV function: ESPVR was adjusted to Ea to evaluate LV/arterial coupling. ESPVR/Ea was numerically lower in the CMET group without statistical difference. PRSW was significantly lower in the CMET group. EDPVR slope as a parameter of diastolic function was significantly steeper in the CMET group. ESPVR, end-systolic pressure-volume relationship; Ea, arterial elastance; PRSW, preload-recruitable stroke work; EDPVR, end-diastolic pressure-volume relationship; LV, left ventricular, *n* = 7 for the CMET, *n* = 8 for the I/R and *n* = 3 for the sham group. ^*^p < 0.05 determined by 1-way ANOVA or Kruskal Wallis test.

The functional parameters measured above depend on the cardiac loading condition. To further characterize the impact of CMET on cardiac function, we employed a method to evaluate load-independent contractility and diastolic function. Using a high-fidelity pressure volume catheter, we evaluated left ventricular pressure-volume relationships. The end-systolic pressure-volume relationship and the preload-recruitable stroke work (PRSW) were calculated as load independent indices of left ventricular contractility. LV/aorta coupling evaluated by the ratio of end-systolic pressure-volume relationship and arterial elastance was numerically lower in the CMET group than I/R group without statistical difference. PRSW was significantly lower in the CMET animals (CMET Group: 25.8 ± 4.0 mmHg, I/R Group: 47.6 ± 6.5 mmHg, sham group: 42.6 ± 7.1, *p* = 0.05). As a measure of left ventricular diastolic function, the end-diastolic pressure-volume relationship (EDPVR) was assessed. The linear EDPVR slope was steeper in the CMET animals (CMET Group: 0.68 ± 0.07 mmHg/ml, I/R Group: 0.40 ± 0.11 mmHg/ml and sham group: 0.2 ± 0.03, *p* = 0.01, Figure 5).

### Coronary Microembolization Leads to Larger Scar Size 1 Week After MI

**Figure 6:**
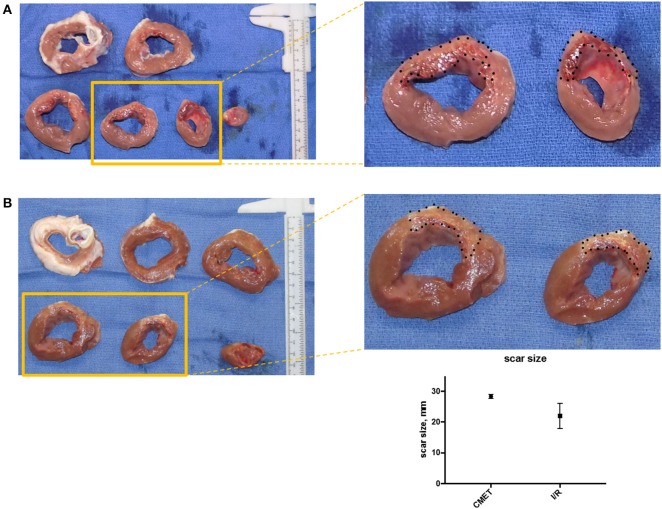
CMET leads to larger scar size compared to I/R. **(A)** Cross-section of the left ventricle from one pig of each group (CMET and I/R). Note the intramyocardial hemorrhage in the apical part of the CMET heart. **(B)** Scar size 1 week after I/R or CMET, *n* = 3. CMET, coronary microembolization using autologous thrombus injection; I/R, ischemia/reperfusion.

Macroscopic examination and scar size measurement was performed in three animals in the I/R group and four animals in the CMET group. The scar size relative to the total left ventricle was numerically larger in the CMET compared to the I/R animals (28.4 ± 0.7 vs. 22.0 ± 4.1%, *p* = 0.25). Representative cross sections of the left ventricle are shown in Figure 6.

In summary, our results demonstrate that autologous thrombus injection in the distal LAD after 60 min of ischemia in pigs results in left ventricular remodeling and impaired left ventricular systolic and diastolic function compared to the I/R model in the subacute phase of an MI.

## Discussion

To our knowledge, this is the first large animal model that closely resembles thrombogenic CME in clinical MI by using autologous clotted blood injection. Our results show that this approach aggravates left ventricular dysfunction when compared to the traditional large animal model of I/R induced MI. This evidence is supported by the functional assessments, including echocardiography, Swan-Ganz catheter, and pressure-volume relationships.

Echocardiographic assessment revealed significant increases in both the systolic and diastolic volumes of the left ventricle after I/R compared to the baseline. The anterior wall showed akinesia and the ejection fraction was reduced. These findings support previous reports that percutaneously induced ischemia followed by reperfusion in pigs impairs cardiac function, similar to the human disease (15, 16). In the current study, we demonstrated that CMET during early reperfusion leads to significant deterioration of cardiac function when compared to the standard I/R model. The impairment of the cardiac function was also evident in invasively measured hemodynamic parameters. One week after MI, Swan-Ganz measurements revealed significantly impaired cardiac output and elevated pulmonary capillary wedge pressure. The impairment of the myocardial contractile function was further demonstrated by load-dependent (maximum left ventricular dP/dt) and load-independent (PRSW) indices. Interestingly, many systolic parameters were not significantly different between the I/R and sham groups, suggesting that the heart after I/R may be able to compensate for infarct-related systolic dysfunction at least at the 1-week time point. This is likely mediated by increasing the contractility of the non-ischemic myocardium. In contrast, both CMET and I/R groups showed significantly impaired diastolic function as evidenced by impaired left ventricular minimum dP/dt, Tau, and steeper EDPVR. Importantly, these parameters were also more impaired in the CMET group than I/R group indicating that both systolic and diastolic functions at 1 week post-MI are more compromised by the CME. Based on our results, it could be that CME delays the infarct healing and prolongs heart failure status compared to the I/R. Further studies with longer follow up are needed to determine whether cardiac function at the chronic stage becomes similar between the I/R and CMET animals.

Scar size at 1 week tended to be larger in the CMET group compared to the I/R group. Lack of statistical difference in scar size may be partially attributed to the small sample size of explanted hearts that were analyzed morphometrically. Differences in size of the ischemic area between the pigs could be another reason, since coronary anatomy is different in each animal with various sizes of diagonal branches. Additionally, the difference in the coronary occlusion time (60 min in the CMET vs. 90 min in the I/R group) might have contributed to the results of the scar size. However, we found complete transmural infarction in CMET animals, which suggests that ischemic area was totally infarcted despite shorter occlusion time. Unfortunately, we were not able to measure the ischemic area-at-risk, because coronary occlusion site was sometimes not evident at 1 week post-MI due to the remodeling of the heart and the coronary artery. Nevertheless, significant functional depression in CMET group highlights negative effects of CME in acute MI.

A deterioration of left ventricular function after CME has been repeatedly shown in other large animal studies (4, 17–19). Our model is unique in inducing CME using autologous thrombus in the setting of preceding ischemia. The majority of the large animal studies of CME employed intracoronary injection of microspheres (4, 18, 20–22). While the microspheres have some advantages (easy handling and dosing), they are chemically inert and therefore not chemoattractant. In addition, they remain in the tissue and do not dissolve unlike microthrombi. The CME material in patients is composed of blood cells (such as platelets, leucocytes etc.) and atherosclerotic material. These materials interact with the vascular wall after embolization and exhibit thrombogenic and inflammatory properties (8). Therefore, both the physical obstruction and the chemoattractant properties of the embolic material play an important role in CME. There are studies that used isolated platelets as a thrombogenic material (17, 19). To our knowledge, this is the first large animal model using the actual thrombus from whole blood.

The method of injection is another issue, which needs to be addressed when inducing CME. In most of the previous studies, the thrombotic material was injected antegrade in the coronary artery. This has the disadvantage of a potential spillover in the off-target myocardium and organs due to thrombus backflow after CMET. Indeed, in our preliminary experiments, thrombus injected into the LAD without coronary balloon occlusion went into the circumflex artery, resulting in embolic infarctions in the circumflex area. Similar off-target infarcts have been reported in several microembolization studies targeting other organs (23, 24). We overcame this issue by breaking up the thrombus and injecting it through the balloon wire lumen while keeping the coronary balloon inflated. This enabled a targeted delivery distal to the balloon-occluded artery and prevented backflow of the thrombus into off-target regions. While we believe the injected thrombi would be eventually absorbed, we were not able to track the fate of injected thrombus. At 1 week, coronary flow assessed by TIMI flow grade remained low in CMET animals with some of them showing grade 0 and 1. This was in contrast to the majority of animals showing TIMI 2 and 3 in the I/R group. However, whether this delay in coronary flow was due to the persistence of initial thrombi or the result of vascular and myocardial remodeling remained unclear.

The third important parameter in our study is the timing of thrombus delivery. We aimed to create a model that can be used to study the acute and subacute mechanisms and effects of CME. Since our objective was to closely recapitulate the clinical condition of thrombogenic CME in myocardial infarction, the injection time point of the thrombus was carefully chosen to be at the end of the ischemic period, just prior to reperfusion. In the majority of previously described animal models, microspheres were injected in the absence of preceding myocardial ischemia. To our knowledge, two large animal studies induced CME in the presence of preceding myocardial ischemia (4, 25). However, in contrast to our model, microspheres were used as thrombotic material. Skyschally et al. (4) used a similar experimental design to investigate the impact of microembolization during early reperfusion. In this study, the authors infused intracoronary microspheres with the onset of reperfusion after 90 min of low-flow ischemia in an open-chest model. Similar to our results, they observed a significant increase in the infarct size compared to the fully reperfused heart, as assessed directly after the procedure. However, microspheres were injected into the whole coronary system including non-ischemic areas in this study. Our model offers higher clinical relevance by injecting the thrombus only into the ischemic coronary in a closed-chest model. Saeed et al. (25) used a closed-chest pig model to induce myocardial ischemia and injected microspheres before reperfusion, using a similar intracoronary injection technique as ours. After a follow up time of 3 days, the investigators observed enhanced patchy microinfarcts in the CME group and impairment of the LV function as measured by magnetic resonance imaging. Despite the differences in the material used to induce CME, functional assessment methods and the observation time of the animals, the functional impairment in these studies is in agreement with our results. The main advantage of our study to the aforementioned is the embolic material used. Clotted blood closely resembles the clinical situation as opposed to chemically inert microspheres. Aside from the mentioned differences, the overall results are in agreement with our findings.

In our study, we observed a higher early mortality in the CMET group. Importantly, while the mortality during the procedure was similar between the groups, the animals in the CMET group exhibited a higher mortality in the first 24 h after the procedure. Since signs of acute heart failure were not evident in the necropsy, we assume that malignant arrhythmias were the cause of deaths. In support of our assumption, pigs are known to be much more susceptible to ventricular arrhythmias during ischemia (26). Previous studies also described higher incidence of malignant arrhythmias in the presence of intracoronary thrombus (27, 28). Future studies with implantable loop recorders may help clarify the cause of death after thrombogenic CME. Whether CME increases arrhythmic events in humans to the similar extent to our pig study remains unclear.

### Significance and Future Implications

In line with the Guidelines for experimental models of myocardial ischemia and infarction (12), our large animal model of CMET in preceding myocardial ischemia simulates the clinical situation of thrombogenic CME during I/R. Impairment of the left ventricular function as observed in our study reflects the clinical findings. Thus, our model is useful in studying mechanisms of thrombogenic coronary thromboembolism and its impact on ensuing infarction. It is also useful for testing new therapies, such as gene and cell therapy, since it allows injection of therapeutic materials without residual foreign bodies remaining in the infarcted artery. Ideal animal model that best reflects the clinical MI with CME would be the one with actual atherosclerosis. However, generating atherosclerosis in pigs is extremely challenging and spontaneous thrombogenic MI events may be rare (29). Our model offers relatively easy induction of thrombogenic CME in pigs. Future studies may involve addition of atherosclerotic materials in the clot to examine the roles of these materials.

### Study Limitations

Modeling a human disease in otherwise healthy animals is an important limitation. This is different from the clinical setting, where the atherosclerotic plaque and its components play an important role in the CME pathophysiology. Furthermore, the thrombus generated in the glass tube does not fully resemble the clinical situation, since the CME material in patients is composed of blood cells (such as platelets, leucocytes etc.) and atherosclerotic materials from the vessel wall. These materials could induce stronger thrombogenic and inflammatory responses compared to our thrombi generated in the glass tube. Additionally, concomitant diseases in humans could contribute significantly to the no-reflow phenomenon due to CME. Our study was likely underpowered in infarct size analysis. Nevertheless, we have characterized the functional difference resulted from the thrombus injection, indicating the importance of thrombogenic CME. Given the high likelihood of arrhythmic deaths in CMET group, we retrospectively think electrophysiologic studies would have added useful information to the study. Lastly, long term observation will be needed to test the durability of the functional impairment and to validate the importance of thrombogenic CME in chronic heart failure.

### Conclusions

Our unique closed-chest pig model of CME using autologous thrombus injection resembles the clinical scenario of CME in myocardial infarction. Exaggerated left ventricular remodeling and impaired function are in line with clinical data. We strongly believe that this model offers various advantages in conducting translational studies for understanding and treating no-reflow associated with thrombogenic CME.

## Data Availability Statement

All datasets generated for this study are included in the article/Supplementary Material.

## Ethics Statement

The animal study was reviewed and approved by Institutional Animal and Use Committee at the Icahn School of Medicine at Mount Sinai.

## Author Contributions

OB, SW, and KI conceived and designed the experiments. OB, ST, KY, AG, SW, and KI performed the experiments. OB and KI analyzed the data. OB, KY, TK, SM, YS, and KI contributed the reagents, materials, and analysis tools. OB, ST, KY, KF, and KI wrote the manuscript. All authors critically revised and approved the final manuscript.

### Conflict of Interest

The authors declare that the research was conducted in the absence of any commercial or financial relationships that could be construed as a potential conflict of interest.

## Supplementary Material

Supplemental Figure 1 Preparation of the autologous thrombus for coronary injection. **1**. Approximately 5–6 ml blood is drawn before any anticoagulants are given to the animal. The blood is quickly transferred to a sterile glass tube. **2**. The glass tube is placed vertically and the blood is allowed to coagulate. **3**. Blood has coagulated. **4**. The thrombus is carefully taken out from the glass tube and placed on a sterile surface. **5–6**. Approximately 1 cm^3^ thrombus is cut. **7**. The thrombus is carefully placed in a 3 ml syringe. Another syringe with 2 ml X-ray contrast is connected via a 3-way connector to the one with the thrombus. Air is carefully removed from both syringes. **8**. The thrombus is thoroughly mixed with the contrast dye until no resistance is encountered in the syringe (see Supplemental Movie 1). **9**. The thrombus-contrast material is injected through the wire lumen of an over-the-wire balloon, while the balloon is still inflated. **10**. The thrombus-contrast mix was placed on a slide. Crushed small thrombi can be found.

Supplemental Movie 1 Preparation of the autologous thrombus for coronary injection.

Supplemental Movie 2 Coronary angiogram of a pig injected with autologous thrombus. The movie shows the LAD flow in two different projections before the balloon occlusion (timecode: 0:00–0:15), the placement of the coronary balloon and the LAD flow during the occlusion (timecode: 0:16–0:25). The last part (timecode 0:26–0:40) depicts the slow flow after reperfusion.
