# Supplementary figures and images for: A Novel Large Animal Model of Thrombogenic Coronary Microembolization
Source: Front Cardiovasc Med. 2019 Nov 5;6:157. doi: 10.3389/fcvm.2019.00157 (PMC6848058; doi:10.3389/fcvm.2019.00157)

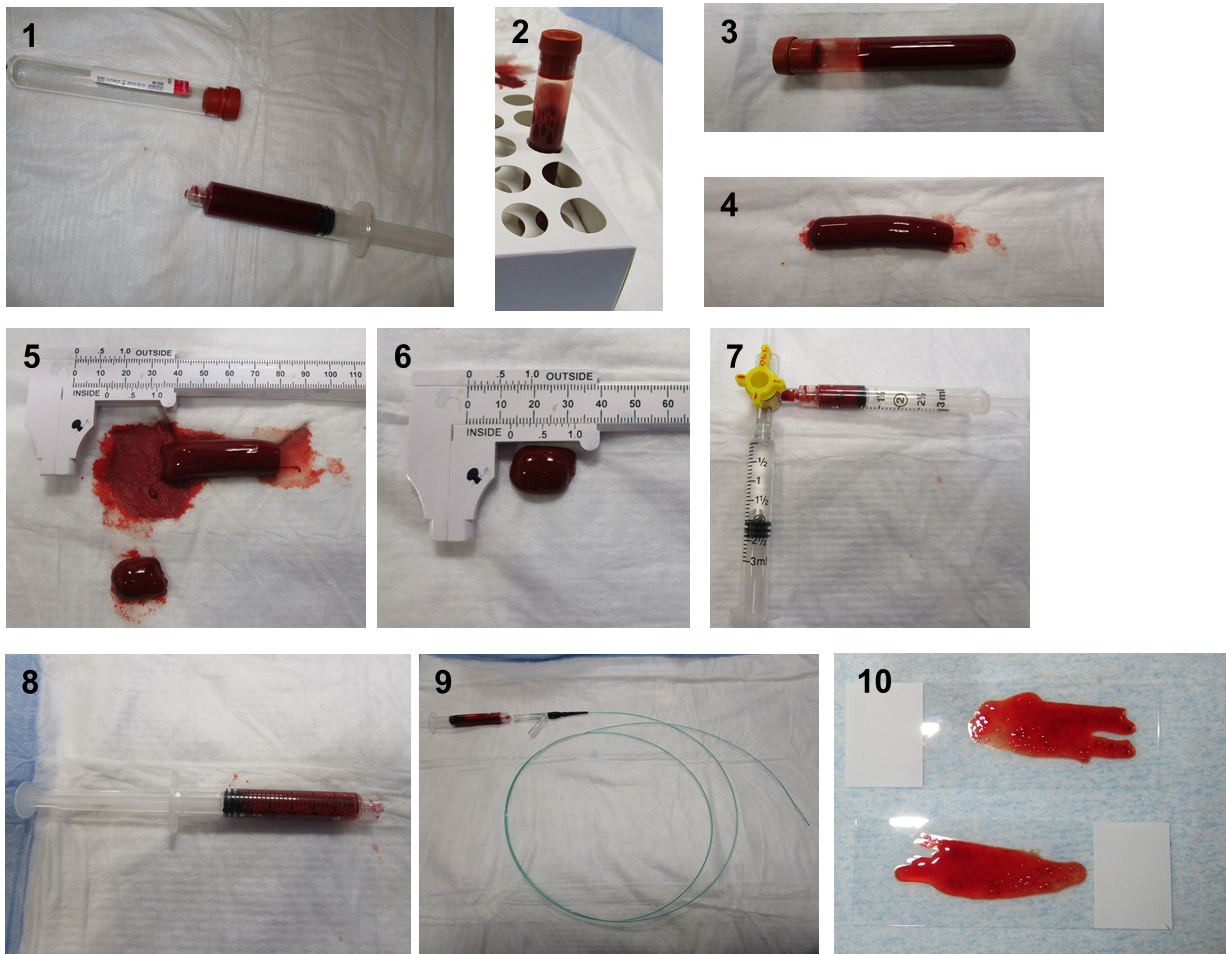

Supplement: Supplemental Figure 1 — Preparation of the autologous thrombus for coronary injection. 1. Approximately 5–6 ml blood is drawn before any anticoagulants are given to the animal. The blood is quickly transferred to a sterile glass tube. 2. The glass tube is placed vertically and the blood is allowed to coagulate. 3. Blood has coagulated. 4. The thrombus is carefully taken out from the glass tube and placed on a sterile surface. 5–6. Approximately 1 cm3 thrombus is cut. 7. The thrombus is carefully placed in a 3 ml syringe. Another syringe with 2 ml X-ray contrast is connected via a 3-way connector to the one with the thrombus. Air is carefully removed from both syringes. 8. The thrombus is thoroughly mixed with the contrast dye until no resistance is encountered in the syringe (see Supplemental Movie 1). 9. The thrombus-contrast material is injected through the wire lumen of an over-the-wire balloon, while the balloon is still inflated. 10. The thrombus-contrast mix was placed on a slide. Crushed small thrombi can be found. [file Image_1.TIF]
